# Large Peripheral Osteomas and Dental Implants: A Case Report

**DOI:** 10.3390/medicina60071181

**Published:** 2024-07-20

**Authors:** Won-Bae Park, Wonhee Park, Joo-An Kim, Seungil Shin, Ji-Youn Hong

**Affiliations:** 1 Private Practice in Periodontics and Implant Dentistry, Seoul 02771, Republic of Korea; wbpdds@naver.com; 2 Department of Prosthodontics, Division of Dentistry, College of Medicine, Hanyang University, Seoul 04763, Republic of Korea; whpark@hanyang.ac.kr; 3 Department of Dentistry, Graduate School, Kyung Hee University, Seoul 02447, Republic of Korea; kjooan@gmail.com; 4 Department of Periodontology, College of Dentistry, Kyung Hee University Dental Hospital, Kyung Hee University, Seoul 02447, Republic of Korea; shin.dmd@khu.ac.kr

**Keywords:** alveolar ridge, benign bone lesion, dental implant, osteoma, peri-implantitis

## Abstract

Peripheral osteoma of the jaw is a rare, benign, slow-growing lesion, which usually appears as a unilateral, pedunculated, radiopaque mass protruding from the periphery and is generally solitary. Multiple osteomas without any syndromic involvement are rare. In the present case, a 75-year-old male patient underwent implant placement in the edentulous posterior ridges of the maxilla and mandible. Over 7 years, multiple masses gradually proliferated in the buccal bone of the implant in three different sextants of the posterior region, reaching a size of 2.0 cm. Clinically and radiologically, these lesions were presumed to be peripheral osteomas and were surgically removed because the large mass made self-performed oral hygiene and maintenance of peri-implant health difficult. The histopathological evaluation confirmed that peripheral osteomas were both compact and cancellous. The patient did not exhibit any other clinical manifestations of Gardner syndrome. Whether dental implant placement and loading are involved in the occurrence of peripheral osteomas is unclear, but they might have affected the consistent growth of the mass as a reactive mechanism. After resection, the functional abilities of chewing and self-cleansing significantly improved. No recurrence of peripheral osteoma was observed after 1 year of follow-up, and peri-implant health was well maintained. Within the limitations of the present case report, multiple peripheral osteomas can occur adjacent to dental implants without any syndromic issues, and a large mass of PO can harm peri-implant health which requires surgical removal. It is speculated that dental implants may be associated with the slow and consistent growth of PO.

## 1. Introduction

Osteomas are benign, asymptomatic, and slow-growing tumors [1] that are primarily observed in the craniofacial bone most frequently in the paranasal sinuses, including the frontal, ethmoid, and maxillary sinuses, while their occurrence in the jawbone is relatively rare [1,2,3]. The age of onset is reported in the range of 14–58 years but usually occurs in 30–40 years, and it has a predilection for men [4,5]. Osteomas are classified into central, peripheral, and exoskeletal types depending on their origins in the endosteum, periosteum, and soft tissue within a muscle, respectively [5,6]. Gardner’s syndrome involves multiple maxillofacial osteomas, in which colorectal polyposis and multiple impacted or supernumerary teeth are associated with a consistent triad [7]. However, uncommonly, multiple osteomas may occur without syndromic compartments. 

Peripheral osteoma (PO) of the jawbone usually appears as a unilateral, pedunculated, radiopaque, mushroom-shaped mass protruding from the periphery [8,9]. Owing to its unique presentation, it is relatively easy to diagnose compared to central osteomas. As mentioned above, the occurrence of PO in the jawbone is uncommon and observed more often in the mandible than in the maxilla [2,6,10]. It appears predominantly at the angle, condyle, and lower border of the mandible. Apart from the maxillary sinus and cases related to Gardner syndrome, maxillary osteomas are rare [5,11,12]. Treatment strategies for POs depend on the size, site, and extent of the lesion, along with its proliferative patterns and clinical manifestations [13]. Asymptomatic lesions of limited size are generally observed on periodic radiographic imaging. When the mass is large and accompanied by progressive growth, facial asymmetry, and functional impairment, surgical intervention for removal is required [13,14,15]. 

Nonetheless, the pathogenesis and etiology of PO remain unclear. Osteomas are true neoplasms, developmental anomalies, or reactive lesions [6,8]; however, PO occurs mostly in adulthood, making it difficult to describe it as a developmental anomaly. Considering its slow growth rate, PO is unlikely to be defined as a true neoplasm. The possibility that PO may be a reactive lesion suggests local trauma, infection, or inflammation as the triggering etiologic factor [1,8,16]. Dental implant placement and advanced surgery, such as bone grafting or maxillary sinus floor augmentation, have been widely used to reconstruct edentulous areas within the jawbones. However, these procedures can cause potent traumatic irritants. Few cases of implant placement at sites with preexisting osteomas have been reported [17,18], and information regarding the occurrence of multiple POs associated with dental implants is limited. The purpose of the present case report was to demonstrate multiple POs in the buccal alveolar bone of both the maxillary and mandibular posterior areas where dental implants were placed and the clinical outcomes related to surgical removal. This case report is described according to the CARE (CAse REport) guidelines [19]. 

## 2. Case Presentation

The patient was a 75-year-old male, a non-smoker who visited a private periodontics and implant dentistry clinic for implant placement in the left maxillary canine and right mandibular first molar teeth. The patient was also requested for the examination of large masses found at the alveolar ridges in both the maxillary and mandibular regions. The patient reported controlled hypertension and hyperlipidemia. No other systemic diseases could compromise the healing process. 

**Figure 1:**
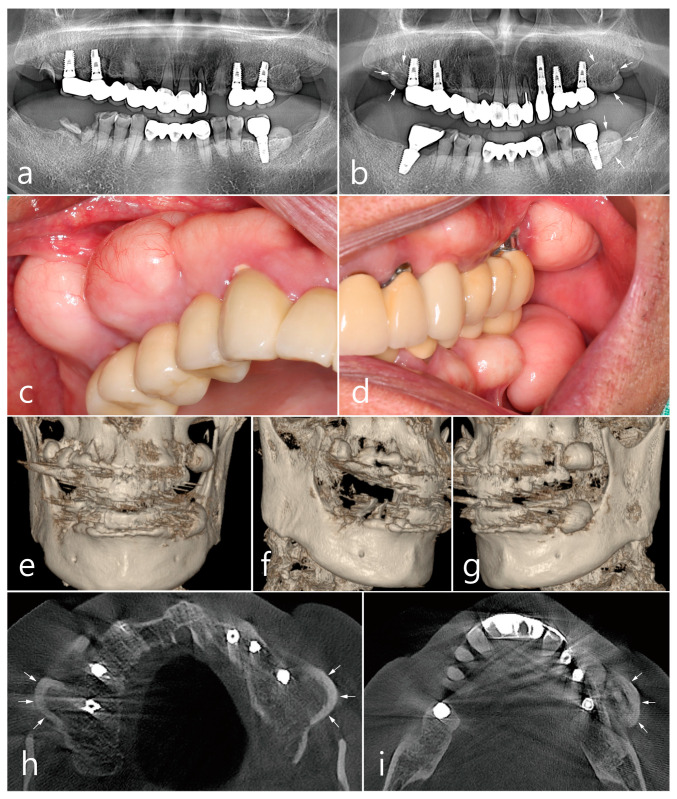
Preoperative examinations. Panoramic radiography (**a**) at the first visit, and (**b**) at one year after two additional dental implants at the maxillary left canine and mandibular right first molar. Ovoid-shaped radiopaque mass (white arrows) shown in the posterior ridges of both the maxilla and left mandible; (**c**,**d**) multiple proliferated round-shaped osteomas observed in the posterior ridges; (**e**) proliferated, well-circumscribed, and pedunculated masses observed in the 3-D images of CBCT; (**f**) a 3-D image of peripheral osteoma (PO) in the maxillary right posterior area; (**g**) a 3-D image of PO occurred in the upper and lower left posterior area; (**h**) an axial image of CBCT showing the proliferated bone mass in the buccal bone of the maxillary posterior implant (white arrow). Mass having external cortical layer and the internal radiolucent marrow space; (**i**) proliferated bone mass in the buccal bone of the mandibular posterior implant (white arrow).

Panoramic radiography performed at the first visit showed that the implants were placed in both the posterior maxillary regions and the first molar of the left mandible. The patient reported that the implants had been loaded for 7 years. Both prostheses in the posterior maxilla were cement-type three-unit bridges and a single screw-type crown was delivered in the left posterior mandible. An assessment of the prostheses showed no specific occlusal overloads or interferences in the posterior implants. Ovoid radiopaque masses were observed in the posterior alveolar ridges where the implants were placed (Figure 1a). The proliferating mass in the maxilla showed a radiolucent medullary space within the outer cortical layer, and the mass in the mandible showed radiopacity with uniform bone density. The root rests of the left maxillary canine and right mandibular first molar were extracted and replaced with dental implants. After 1 year of prosthetic loading on the two additional implants, the panoramic view showed a more pronounced radiopaque mass in the three previous areas (Figure 1b). 

Prominent dome-shaped masses were observed during the patient’s intraoral clinical examination (Figure 1c,d). The patient experienced difficulties with tooth brushing and mastication. The patient said that he was not aware of the appearance of the bone mass when the implant was first placed and that he was able to recognize it only after it grew over time. No paresthesia or tenderness was associated with the lesion, and the regional lymph nodes were not palpable. Based on clinical and radiological findings, the bone mass in this case was diagnosed as PO. The implant adjacent to the PO was accompanied by peri-mucositis, and peri-implant bone loss with thread exposure was observed in the left maxillary first molar implant. Proliferating, well-circumscribed, and pedunculated masses were clearly observed in the 3-D cone-beam computed tomography (CBCT) (Figure 1e–g). On the axial CBCT image, the PO proliferated from the buccal bone of each maxillary posterior region (white arrows) (Figure 1h). The mass was lined with a dense external cortical layer and internal radiolucent bone marrow. PO of the buccal bone of the left mandibular posterior implant (white arrows) showed a dense and thick outer cortical layer and a relative lack of bone marrow space (Figure 1i).

### 2.1. Left Maxillary Posterior Region

**Figure 2:**
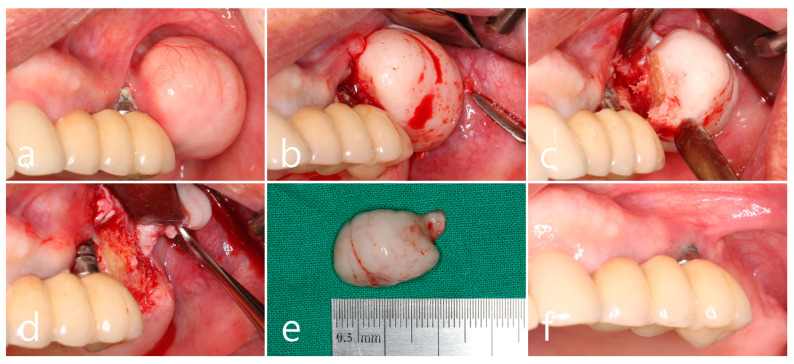
Maxillary left posterior region. (**a**) A large mass of peripheral osteoma (PO) at the buccal alveolar bone adjacent to the first molar implant. Mucosal recession and plaque retention are shown at the implant; (**b**) flap reflection and exposure of the PO; (**c**) osteotomy and separation at the base of the pedunculated PO using a surgical round bur, and a bone chisel; (**d**) exposure of the implant thread with buccal bone dehiscence; (**e**) the removed specimen in a size of 1.8 × 2.0 cm; (**f**) maintenance after 1 year of follow-up.

In the left maxillary posterior region, the growth of PO from the buccal surface of the ridge almost reached the occlusal table of the prosthesis adjacent to the first molar implant, which increased the discomfort of the patient and disturbed self-performed oral hygiene. Progressive crestal bone loss, plaque retention, chronic inflammation, and marginal mucosal recession around the implant were observed during the follow-up period (Figure 2a). Surgical intervention was planned to remove PO. Under local anesthesia, a mucosal incision was made to include PO with adjacent implants, and a full-thickness flap was reflected. Moreover, PO surrounded by the cortical layer was exposed (Figure 2b). Osteotomy using a round surgical bur and bone chisel was performed to separate the base of the pedunculated hard tissue mass from the buccal bone surface (Figure 2c). The exposed surface of the ridge was smoothened using a bone file. A peri-implant dehiscence defect with a height of 3 mm was observed on the first molar implant (Figure 2d). The removed specimen was 1.8 × 2.0 × 1.0 cm in size and consisted of loose cancellous bone within the cortical layer (Figure 2e). Healing of the surgical site was uneventful, and the reduced buccal contour and ridge volume were maintained until after 1 year of follow-up. Reduced pocket depth without bleeding on probing was observed with adequate plaque control (Figure 2f).

### 2.2. Left Mandibular Posterior Region

**Figure 3:**
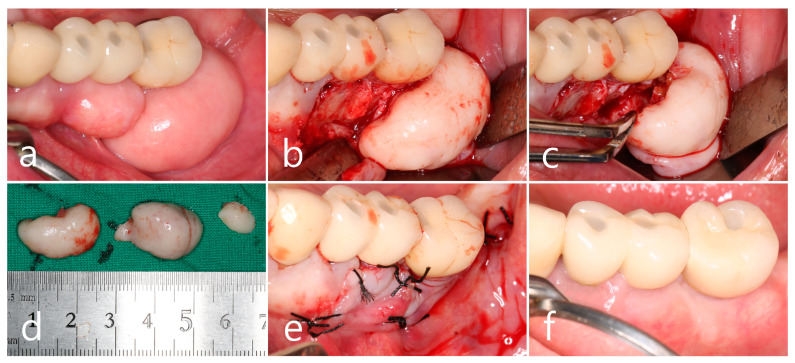
Mandibular left posterior region. (**a**) Multiple masses of PO at the buccal alveolar ridge of the left mandibular posterior implants; (**b**) flap reflection and exposure of the PO; (**c**) osteotomy at the base of PO using a round bur and a bone chisel; (**d**) the specimens removed from the upper and lower jaws; (**e**) flap closure; (**f**) maintenance after 1 year of follow-up.

In the left mandibular posterior area, multiple well-defined round masses of PO were observed on the buccal ridge surface from the premolar to molar regions (Figure 3a). PO removal was performed as described above (Figure 3a). The buccal mucoperiosteal flap was reflected under local anesthesia (Figure 3b). Osteotomy was performed at the base of PO using a round surgical bur and separated using a bone chisel. However, the cortical layer of the PO was thick and difficult to remove compared to the maxillary sites (Figure 3c). The size of the largest specimen removed from the mandible was about 1.5 × 2.0 × 1.0 cm (Figure 3d). The specimens were removed from the left maxillary and mandibular areas and fixed in 10% formalin for histological examination. The exposed alveolar ridge was smoothened with a bone file, and the flap was closed (Figure 3e). A well-healed surgical site was observed 1 year postoperatively (Figure 3f).

**Figure 4:**
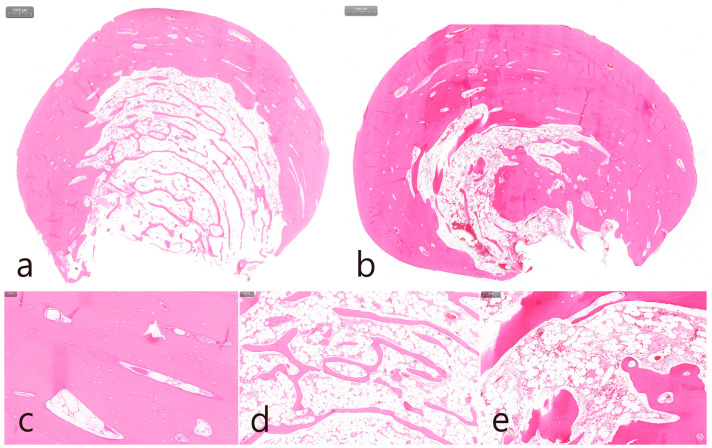
Histologic features (H-E stain). (**a**) Specimen from the maxillary left posterior region with cancellous-type PO. Oval-shaped mass composed of mature compact bone in the outer layer and fatty marrow space filled with adipose, vascularized connective tissue, and thin trabecular bones in the inner core; (**b**) specimen from the mandibular left posterior region with compact-type PO. Oval-shaped mass composed of thick, dense lamellar bone and a sparse fatty marrow space; (**c**) outer layer of mature lamellar bone commonly found in both specimens; (**d**) medullary space containing numerous adipose tissues and loosely arranged thin trabecular bone in the cancellous-type PO; (**e**) medullary space containing external lamellar bone proliferated inward rather than trabecular bone spicules in compact-type PO.

Histopathological examination of the specimen revealed an oval bone mass (Figure 4a,b). The specimen from the maxillary posterior region had a compact layer of mature lamellar bone surrounding the outer surface of the mass, and the inner portion was composed of fatty bone marrow space filled with adipose tissue, vascularized connective tissue, and cancellous bone spicules. This is a cancellous PO (Figure 4a). The cortical layer of the mandibular specimen was thicker than that of the maxillary specimen (Figure 4b). The cortical layer was composed of dense lamellar bone and showed a compact PO (Figure 4b,c). Highly magnified histological views confirmed that the cancellous-type PO had a medullary space filled with loose connective tissue mainly composed of fatty tissue, and thin bone spicules were sparsely arranged (Figure 4d). In the compact-type PO, the medullary space occupied little space in the inner portion, and the lamellar bone proliferated inward rather than the bone spicules (Figure 4e).

### 2.3. Right Maxillary Posterior Region

**Figure 5:**
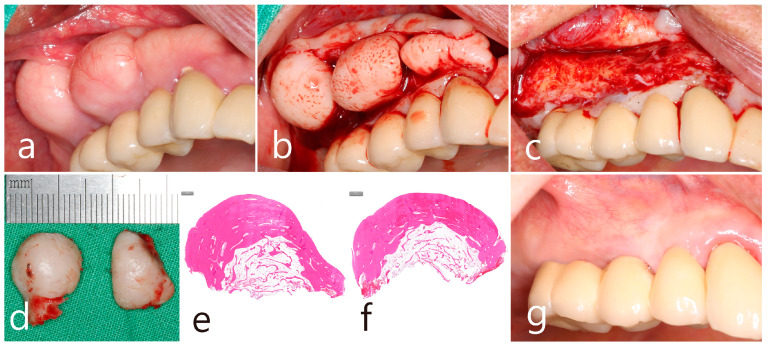
Maxillary right posterior region. (**a**) PO widely located on the buccal side of the ridge in the maxillary posterior area adjacent to the teeth and implants; (**b**) flap reflection and exposure of multiple isolated POs; (**c**) removal of the mass and smoothening of the bone surface; (**d**) the removed specimens in size approximately of 1.0–1.5 cm; (**e**,**f**) histologic features of cancellous-type PO in both specimens; (**g**) maintenance after 1 year of follow-up.

**Figure 6:**
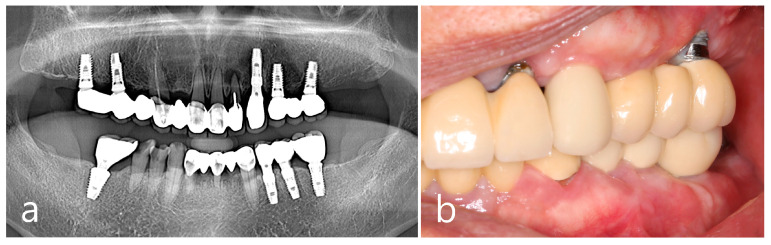
Examination after one year of the surgery. Panoramic view (**a**) and intraoral clinical view (**b**) showing no recurrence of PO.

The removal of the PO in the right posterior maxilla was planned 2 months after surgery in the left quadrant. Multiple POs arising from the buccal alveolar ridge adjacent to the posterior implant were observed (Figure 5a). After the flap was reflected, three isolated POs were observed (Figure 5b) and removed via osteotomy at the base of the pedunculated masses. The exposed surface was smoothened using a bone file (Figure 5c). The size of the largest specimen was approximately 1.5 × 1.0 × 1.0 cm (Figure 5d). Histologically, the specimen was a cancellous PO with a wide medullary space filled with fatty and loose connective tissue surrounded by a compact cortical layer (Figure 5e,f). A well-healed surgical site was observed after 1 year of follow-up (Figure 5g). No signs of recurrence were found in the clinical and radiographic images taken 1 year after surgery (Figure 6a,b).

## 3. Discussion

This case report showed multiple POs that occurred at the buccal bone of the alveolar ridges in the posterior areas of both the maxilla and mandible, where the rehabilitation of edentulous areas with dental implants was performed. Prosthetic loading was performed for more than 7 years, and the bone masses seemed to have grown at a slow rate. According to the patient’s report, he incidentally found bone masses several years after prosthetic delivery and was unaware of their presence when the implants were first placed. It is not clear whether there was a small mass of preexisting PO before the surgery, as baseline records were not available; therefore, it was difficult to determine whether implant placement played a causative role in the occurrence of PO. However, dental implants may be involved in the progressive growth of hard-tissue masses as reactive lesions. 

Radiographic imaging of PO shows an oval-shaped, radiopaque, well-circumscribed mass with a pedunculated or wide base attached to a normal cortical bone [5]. Traditional radiographs, such as panoramic and Waters’ views, are usually obtained during periodic follow-up evaluations; however, additional CT or CBCT with 3-D reconstructed images might help localize the lesion with better resolution. Although not routinely used, a bone scan can be considered to disclose the physiologic activity of the PO and determine whether the mass is a long-standing mature lesion or a young, actively growing lesion [1,5]. In addition to radiographic examinations using a panoramic view and CBCT, histopathological evaluation of the bone mass specimens was performed after surgical removal. Histologically, osteomas are characterized by the atypical proliferation of compact or cancellous bone and the deposition of osteoblasts. PO can be classified into three types: compact, cancellous, and mixed [5,20,21]. The compact type is an osteoma comprising normal mature dense bone with minimal marrow spaces and occasional Haversian canals. The cancellous type usually contains trabecular bone with a large portion of fibro-fatty marrow and a surface with a thin cortical layer. In the present case, both types of POs were found in a patient with relatively distinct aspects of the maxilla and mandible, which seemed to resemble the bone of origin. POs in the left mandibular posterior region had compact histologic features, which made them difficult to remove because of the hardness at the margins to resect. In contrast, maxillary POs in both posterior areas were determined to be cancellous types and were relatively easy to excise compared to mandibular lesions. Although conventional instruments, such as surgical round burs and bone chisels were used for ostectomy, piezosurgery could also be considered, which is advantageous for less trauma to soft and hard tissues and improved accessibility [17,22]. 

Lesions that need to be differentially diagnosed from PO include exostoses and several inflammatory and neoplastic lesions, such as sclerotic patterns of chronic osteomyelitis, periosteal osteoblastoma, peripheral ossifying fibroma, parosteal osteosarcoma, and osteoid osteoma [6,23,24]. It is difficult to distinguish between exostoses and POs of the jaws. Clinically, exostoses are usually multiple and located within the attached gingival area [25]. It is likely that exostoses start growing in the prepubescent phase and that the growth rate decreases or ceases when maturity is reached [20,25]. Solitary exostoses are rare and may be associated with local trauma or intraoral soft tissue surgeries, such as gingival and cutaneous grafts. Exostoses and POs present similar histopathological features but osteomas may have a greater portion of the medullary cavity according to the subtype [26,27]. The present case showed multiple bone masses, which are rare in POs, and involved both compact and cancellous types. Slow and continuous growth patterns without symptoms were also considered in the diagnosis of PO. In addition, patients with multiple osteomas should be evaluated for Gardner’s syndrome [7]. Gardner’s syndrome is an inherited autosomal dominant condition characterized by colorectal polyposis, cutaneous sebaceous cysts, and supernumerary teeth, which was not the case in the present patient having no specific gastrointestinal and dental anomaly. However, certain reports show multiple craniofacial osteomas without syndromic involvement [28,29]. 

In the literature, limited information is available on POs occurring adjacent to dental implants and their clinical outcomes. Certain studies have reported the clinical results of dental implantation in preexisting hyperdense jaw lesions, including central osteoma, osteoid osteoma, odontoma, cementoblastoma, idiopathic osteosclerosis, and condensing osteitis [17,18,30]. Due to the lack of baseline data regarding the preexisting osteomas, dental implants as causation of POs are inconclusive in this case report. However, the patient’s report on the incidental notification of the mass after the prosthetic loading and the overgrowth mass partly covering the crown portion may give rise to the possibility that slow additional bone growth in response to trauma during implant surgery and functional loading occurred. The reactive mechanism to trauma or infection is also supported by the finding that the angle and lower border of the mandibular body are the most common sites of POs in the jawbones, where the sites are more susceptible to trauma. Muscle traction may also play an important role because the PO of the jaw is usually located close to the area of muscle attachment [6,8]. In this case, POs occurred in a limited manner in three sextants of the maxillary and mandibular posterior regions where the dental implants were exposed to chewing forces and loading function. POs were not observed in the right posterior mandible, where the molars were missing. In previous studies, overload on most posterior implants has been suggested to affect the damage to the periodontal apparatus in adjacent and antagonistic teeth, resulting in tooth loss [31,32]. Although there were no clinical signs of mechanical overloads and occlusal interference of the implant prosthesis during the oral examination in this case, implants may be subjected to heavier loading forces, and the trauma from occlusion applied to the peri-implant tissue could be a potent irritant that can induce consistent growth. In this sense, the ridge surrounding the latest first molar implant in the right mandible should also be carefully monitored in a long-term follow-up. 

The large PO mass rendered the intraoral condition inappropriate for self-maintained oral hygiene. The patient showed prominent plaque retention around the mucosal margins of the posterior implants, accompanied by peri-implant mucositis. In the left maxillary posterior region, the first molar implant was involved in peri-implantitis, which involved crestal bone loss, mucosal recession, and exposure of the fixture threads. Buccally protruding PO in the proximity of the implant at the distal surface made it difficult to access with a toothbrush or interdental cleansing aids. Consistent peri-implant mucositis and peri-implantitis may adversely affect the progression of PO with respect to infection and inflammation. After surgical removal of the POs, the buccal contour of the alveolar ridge adjacent to the posterior implants became appropriate for self-cleaning and maintenance. The recurrence of PO after excision has been reported to be extremely rare [1,24]. 

This study has some limitations. When the patient visited the clinic, posterior implant rehabilitation of both the posterior maxilla and left posterior mandible had already been performed, and baseline records, including the condition of the bone or preexisting POs before implant placement, were not available. The patient incidentally recognized the presence of POs after a few years of loading, and the timing of the occurrence was not clear. Therefore, the causative factors for the incidence and growth of POs cannot be conclusively established. Other factors contributing to POs such as genetic predispositions and environmental influences should be further evaluated through similar clinical cases with long-term outcomes. In addition, the recurrence and complication of PO after surgical removal cannot be fully suggested as the present study shows only short-term clinical outcomes and further long-term observation of the case is necessary. Despite this limitation, this case report demonstrated that multiple POs occurred in the jawbones of both the maxilla and mandible close to the posterior implants, which is very rare. Dental implant loading could be associated with consistent growth of the mass, and large POs may negatively affect peri-implant health which requires removal. 

## 4. Conclusions

Within the limitations of the present case report, multiple POs can occur adjacent to dental implants without syndromic problems, and a large mass of PO can harm peri-implant health which requires surgical removal. It is speculated that dental implants may be associated with the slow and consistent growth of POs.

## Data Availability

The data are contained within the article.
